# Supplementary material for: “He who pays the piper calls the tune”: Researcher experiences of funder suppression of health behaviour intervention trial findings
Source: PLoS One. 2021 Aug 18;16(8):e0255704. doi: 10.1371/journal.pone.0255704 (PMC8372890; doi:10.1371/journal.pone.0255704)
Supplement: S1 Table — (DOCX) [file pone.0255704.s001.docx]

**S1 Table. Characteristics of responders and non-responders.**

| **Characteristics of trial** | **Responders n = 104 (%)** | **Non- responders n = 104 (%)** |
| --- | --- | --- |
| Age of publication 2007-2016 (mean, SD) | 6.45 (2.48) | 6.77 (2.37) |
| Study design |  |  |
| RCT | 86 (83%) | 77 (74%) |
| Controlled trial | 18 (17%) | 27 (26%) |
| Health behaviour targeted |  |  |
| Nutrition/Physical activity | 57 (55%) | 49 (47%) |
| Sexual risk/substance use | 47 (45%) | 55 (53%) |
| Country |  |  |
| Europe | 34 (33%) | 28 (27%) |
| North America | 35 (34%) | 54 (52%) |
| Oceania | 18 (17%) | 6 (6%) |
| Other | 17 (16%) | 16 (15%) |
| Full democracy |  |  |
| Yes | 63 (61%) | 58 (56%) |
| No | 16 (15%) | 14 (13%) |
| Missing | 25 (24%) | 32 (31%) |
| Funding |  |  |
| Other Government Agency | 43 (39%) | 51 (49%) |
| Independent funding | 29 (28%) | 22 (21%) |
| Industry | 6 (6%) | 5 (5%) |
| Philanthropic | 10 (10%) | 9 (9%) |
| Multiple/unknown | 10 (10%) | 11 (11%) |
